# miR-192 inhibits the activation of hepatic stellate cells by targeting Rictor

**DOI:** 10.1515/med-2023-0879

**Published:** 2023-12-26

**Authors:** Hui Kang, Jie Luo, Chun Wang, Yinghui Hong, Mingliang Ye, Yang Ding, Qiu Zhao, Ying Chang

**Affiliations:** Department of Gastroenterology, Zhongnan Hospital of Wuhan University, Wuhan 430071, China; Hubei Clinical Center and Key Laboratory of Intestinal and Colorectal Diseases, Wuhan 430071, China

**Keywords:** liver fibrosis, microRNA-192, Rictor, hepatic stellate cell, Smad

## Abstract

The activation of hepatic stellate cells (HSCs) is regarded as the primary driving factor of liver fibrosis. miR-192, a miRNA associated with hepatocellular carcinoma and enriched in HSCs, has an undisclosed role in HSC activation and liver fibrosis. In this study, a CCl_4_-induced rat liver fibrosis model and transforming growth factor-beta 1 (TGF-β1)-treated HSC lines (LX-2 and HSC-T6) were used to detect miR-192 and Rictor levels *in vivo* and *in vitro*. Bioinformatic analysis and a dual luciferase assay were used to predict and confirm the interaction of Rictor with miR-192. Gain- and/or loss-of-function methods evaluated molecular changes and HSC activation phenotypes, detected by quantitative real-time PCR, western blotting, and immunofluorescence. We observed a gradual downregulation of miR-192 and upregulation of Rictor during CCl_4_-induced liver fibrosis/cirrhosis in rats. Enriched miR-192 was downregulated, while Rictor was upregulated in TGF-β1-activated HSCs. miR-192 inhibited the activation of HSCs by directly targeting Rictor. High miR-192/low Rictor expression attenuated the fibrotic-related gene expression by AKT/mTORC2 signaling. In conclusion, miR-192 could inhibit the activation of HSCs by directly targeting Rictor in the AKT/mTORC2 signaling pathway. This study provides insights into potential therapeutic targets for liver fibrosis and cirrhosis.

## 1 Introduction

Liver fibrosis is a complex pathological protective response to liver injury caused by various reasons. Without effective treatment, liver fibrosis might deteriorate to cirrhosis, which resulted in over 2 million deaths in 2017 worldwide [1]. However, fibrosis is generally considered regressive and even reversible [2]. Therefore, besides the therapies for its underlying causes, the development of effective treatment targeting fibrosis directly is also significant.

Hepatic stellate cells (HSCs) represent 10–15% of all resident liver cells [3,4]. HSC was first reported as “Sternzellen” by von Kupffer in 1876 and got the consensus term “hepatic stellate cell” in 1995. It has been identified that HSC is the major fibrogenic cell and plays its key role in liver fibrosis by its transdifferentiation called “activation” – after the triggers of intercellular or extracellular stimuli, the quiescent, vitamin-A-storing HSCs transform into myofibroblast-like ones, exerting subsequent phenotypic changes, including proliferation, contractility, and fibrogenesis. Some proteins, including α-SMA (alpha-smooth muscle actin, a marker of myofibroblast formation) and COLIA1 (collagen type I alpha1, the major component of type I collagen), have been recognized as markers for HSC activation. Activated HSCs secrete large amounts of collagen and thus form scar tissue, which is a self-repair process within damaged livers that ultimately leads to chronic fibrosis or cirrhosis [4,5].

In addition to collagen production, activated HSCs secrete transforming growth factor-beta 1 (TGF-β1), the most potent fibrogenic cytokine, which further accelerates the activation of HSCs, creating a feedback loop that exacerbates the fibrotic process [4,6]. TGF-β-driven fibrosis can be mediated through Smad-dependent and non-Smad pathways [7]. Although the classical TGF-β signaling pathway is Smad mediated, the activated receptors also work through other signal transducers, for instance, the mitogen-activated protein kinase pathways, the IkB kinase (IKK), phosphatidylinositol-3 kinase (PI3K), Akt, and Rho family GTPases [8].

mTOR (the mammalian target of rapamycin) is a target of the Akt kinase. Rictor has been widely known as a subunit of mTORC2 (the mammalian target of rapamycin complex 2). Unlike mTORC1, which contributes mainly to cell growth and metabolism, mTORC2 has comprehensive biological functions involved in cell growth, proliferation, survival, cytoskeletal remodeling, and migration, which require the participation of Rictor [9,10]. Till now, Rictor’s involvement in numerous cancer cell types, including those in the prostate, lung, breast, and liver cancer [11,12], has begun to be unraveled. Recent investigations have reported the profibrotic effects of mTORC2/Rictor in kidney [13], lung [14], and liver fibrosis [15]. Nevertheless, the precise role of Rictor in liver fibrosis, especially its biological function in HSC, still needs to be further explored.

microRNAs (miRNAs) are short ∼22 nucleotide RNAs that could directly regulate target mRNAs post-transcriptionally, which help sculpt the expression of most mRNAs in diverse eukaryotic lineages [16]. Among the extensive array of miRNAs, a study about quiescent HSCs piqued our interest, as it revealed an early down-regulation of miR-192 during the onset of fibrosis [17]. Although miR-192’s inhibitory role in epithelial–mesenchymal transition (EMT, which is implicated in HSC activation) was reported in both kidney and liver disease [18,19,20], the function and mechanism by which miR-192 represses the activation of quiescent HSC have yet to be illuminated.

Therefore, we hypothesize that miR-192 plays a dynamic role in the progression of liver fibrosis and cirrhosis. Our research aims to investigate the functions of miR-192 and its interaction with Rictor in the activation of HSCs.

## 2 Materials and methods

### 2.1 Cell culture

The human HSC cell line LX-2 was purchased from the Cell Center of Shanghai Institutes for Biological Sciences, and the rat HSC-T6 cell line was purchased from the China Center for Type Culture Collection of Wuhan University. LX-2 and HSC-T6 cells were cultured in Dulbecco’s Modified Eagle’s Medium (HyClone, USA) and supplemented with 10% fetal bovine serum (Gibco, Thermo Fisher Scientific, USA) and 1% penicillin/streptomycin (HyClone, USA) at 37°C with 5% CO_2_.

### 2.2 miRNA mimic, siRNA, plasmid, and cell transfection

miR-192-5p mimic, siRNA of Rictor, and negative controls were purchased from Guangzhou RiboBio, China. myc-Rictor corrected was a gift from David Sabatini (Addgene plasmid # 11367; http://n2t.net/addgene:11367; RRID: Addgene11367). Cells were transfected by using Lipofectamine 2000 (Invitrogen, Carlsbad, CA, USA) according to the manufacturer’s instructions.

### 2.3 RNA extraction, reverse transcription, and qRT-PCR

The total RNA of cells and tissues was extracted by Life Trizol (Ambion, USA) according to the instructions. The quality and concentration of RNA were detected by NanoDrop 2000 (Thermo Fisher Scientific, USA) and cDNA was reversed transcribed with the ReverTra Ace^®^ quantitative real-time PCR (qPCR) RT Kit (Toyobo, Osaka, Japan). Bulge-Loop™ miRNA RT-PCR Primer Sets and U6 primers were purchased from Guangzhou Ribobio, China. qRT-PCR was performed by UltraSYBR Mixture (CWbio, Beijing, China). The relative levels of miRNAs and genes were normalized by U6 and GAPDH separately. The gene primers (Tsingke, Wuhan, China) are listed in Table 1.

**Table 1:** Primer sequences for PCR

Gene	Primer sequence
(Homo) ACTA2^1^ F	GGGAATGGGACAAAAAGACA
(Homo) ACTA2 R	CTTCAGGGGCAACACGAA
(Rattus) Acta2 F	ATGACCCAGATTATGTTTGAGACC
(Rattus) Acta2 R	CCAGAGTCCAGCACAATACCA
(Homo) COL1A1 F	TGTGCGATGACGTGATCTGTGA
(Homo) COL1A1 R	CTCGACGCCGGTGGTTTCTT
(Rattus) Col1a1 F	GGGCAAGACAGTCATCGAATACA
(Rattus) Col1a1 R	CAGATTGGGATGGAGGGAGTTTA
(Homo) RICTOR F	AGCTCACGGTTGTAGGTTGC
(Homo) RICTOR R	TTGAAGACTTCTTTCGGGTT
(Rattus) Rictor F	CCGCTCATGGGCAGGTATTA
(Rattus) Rictor R	CAACAGGCAGAGGGAGACGA
(Homo) SMAD3 F	GTTGGTGGAGGGTGTAGTGG
(Homo) SMAD3 R	GGCTTCTTGGATAGATGGCTC
(Rattus) SMAD3 F	CCAGTGCTACCTCCAGTGTTG
(Rattus) SMAD3 R	TCTGGTGGTCGCTAGTTTCTC
(Homo) GAPDH F	GGAAAGCCTGCCGGTGACTA
(Homo) GAPDH R	CGCCCAATACGACCAAATCA
(Rattus) Gapdh F	CGGCAAGTTCAACGGCACAG
(Rattus) Gapdh R	CGCCAGTAGACTCCACGACAT

1: The α-smooth muscle actin is also known as ACTA2.

### 2.4 Luciferase assay

LX-2 cells were cultured at a density of 5 ×  10^4^ cells/well in 24-well culture plates and co-transfected with 2 μg of pmirGLO-RICTOR-wt/pmirGLO-RICTOR-mut, and 50 nM final concentration of miR-192 mimics/miR-NC. Twenty-four hours post-transfection, luciferase activity was measured using the Dual-Luciferase® Reporter Assay System (# E2920, Promega, USA) and Enspire 2300 (PerkinElmer). Normalized firefly luciferase activity (firefly luciferase activity/Renilla luciferase activity) for each construct was compared to that of the pmirGLO Vector no-insert control. For each group, luciferase activity was tested in quintuplicate.

### 2.5 Immunofluorescence staining

Transfected LX-2 cells were fixed with 4% paraformaldehyde for 15 min. After three washes with phosphate-buffered saline (PBS), cells were blocked for 1 h at room temperature (1× PBS/1% BSA/0.3% Triton X-100). After three washes with PBS, add the primary antibody (1:200, vimentin, # 5741; CST) to a 12-h incubation at 4°C. After another three washes with PBS, a fluorescent secondary antibody (1:500, Cy3, GB21303, Servicebio) was applied and incubated for 2 h at room temperature; After three washes with PBS, nuclear staining with DAPI was performed for 5 min. After three times of PBS washes, cells were observed and photographed under a fluorescence microscope. Statistical significance was calculated as per the intensity of red colors using ImageJ software.

### 2.6 Protein extraction, antibodies, and western blotting

The total protein of tissues and cells was extracted using RIPA Lysis Buffer (Beyotime Institute of Biotechnology, Jiangsu, China) with protease inhibitor Phenylmethylsulfonyl fluoride (1:100, Servicebio, Wuhan, China) and phosphatase inhibitor cocktail (1:25, MedChem Express, USA). The protein concentration was detected and quantified by the Enhanced Bicinchoninic Acid (BCA) Protein Assay Kit (Beyotime Institute of Biotechnology). Equivalent total protein (35 mg) after denaturation in a water bath at 100°C was separated by electrophoresis with 8–15% sodium dodecyl sulfate–polyacrylamide gel electrophoresis gels and transferred to polyvinylidene fluoride membranes (Millipore, USA). The membranes were blocked using 5% skim milk solution for 2 h at room temperature. After incubated with primary antibodies overnight at 4°C and subsequent secondary antibodies for 1.5 h at room temperature, signals of the strips were visualized using an enhanced ECL Assay kit (Servicebio, Wuhan, China) and detected using GENESys (Synoptics Ltd., UK). Antibodies used in this study were listed as follows. Rabbit antibodies of α-SMA (1:1,000, #19245), phospho-Smad3 (Ser423/425) (1:1,000, #9520), Smad3 (1:1,000, #9513), Rictor (1:1,000, #2114), collagen I A1 (1:1,000, #84336), p-mTOR (Ser2448) (1:1,000, #5536), p-AKT (Ser473) (1:1,000, #4060), and Vimentin (1:1,000, #5741) were provided by Cell Signaling Technology (USA). Abcam provided another kind of phospho-Smad3 (1:2,000, ab52903) antibody. The mouse antibody of Gapdh was provided by Sanying Biotechnology (China). The secondary antibodies were goat anti-mouse IgG-horseradish peroxidase (1:3,500, #A25012, Abbkine) and goat anti-rabbit IgG-horseradish peroxidase (1:3,500, #A23220, Abbkine). Densitometric analysis of western blotting results was performed by ImageJ.

### 2.7 Animal experiments

Wistar rats (male, 7–8 weeks old, 200–220 g) were purchased from Beijing HFK Bioscience Co., Ltd. Experiments were performed under a project license (No.: 2017055) granted by the Institutional Animal Care and Use Committee of Wuhan University, in compliance with the Center for Animal Experiment/Animal Biosafety Level-III Laboratory of Wuhan University Guidelines for the care and use of animals.

Twenty-two rats were randomly divided into two groups: the control group (*n* = 4) and the carbon tetrachloride (CCl_4_) group (*n* = 18). Liver fibrosis/cirrhosis model was constructed by intraperitoneal injection of 40% CCl_4_ dissolved in maize oil (1.5 mL/kg, twice a week) for at least 8 weeks. Then, five to six rats including one belonging to the control group were sacrificed every two weeks until 16 weeks. The liver tissues were immediately stored in RNAStore (TiangenBiotech, Beijing, China) or fixed in 4% paraformaldehyde for subsequent experiments. Hematoxylin and eosin (H&E) staining and Masson’s trichrome staining were performed by Servicebio Company (Wuhan, China) and observed by an inverted microscope (Olympus IX3). Liver fibrosis stages (F1–F4) were evaluated by experienced investigators according to the Metavir system [21].

### 2.8 Statistical analysis

Independent experiments were repeated three times or more. The data are represented as the mean and standard deviation. GraphPad Prism 8 software (GraphPad, USA) was used to assess statistical analyses. *P* < 0.05 was considered significant (*P < 0.05, **P < 0.01, ***P < 0.001).

## 3 Results

### 3.1 miR-192 downregulated and Rictor upregulated gradually in CCl_4_-induced rat liver fibrosis

**Figure 1:**
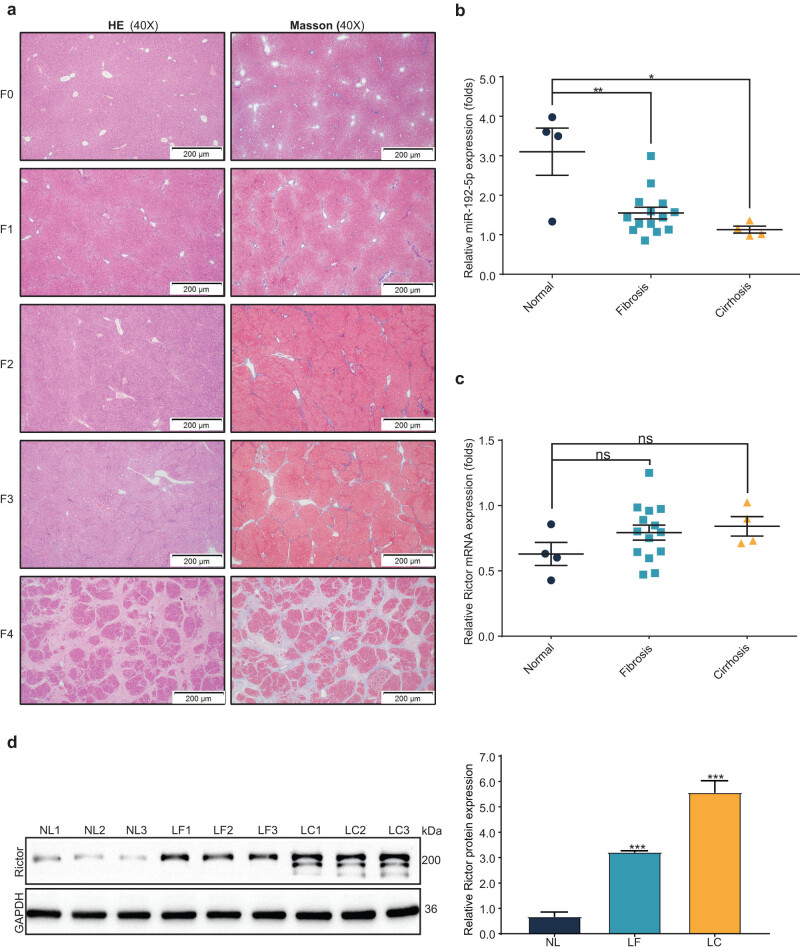
miR-192 downregulated and Rictor upregulated gradually in CCl_4_-induced rat liver fibrosis (a) Representative H&E and Masson’s trichrome-stained images (×40). Liver fibrosis stages (F0–F4) were evaluated by experienced investigators according to the Metavir system. Cytoplasm and erythrocytes were presented as red; collagen was presented as blue in Masson images. miR-192 (b) and Rictor (c) expression was measured by qRT-PCR in normal (F0, *n* = 4), fibrosis (F1-3, *n* = 14), and cirrhosis (F4, *n* = 4) samples. Each dot indicates the expression level of an individual case, calculated by the 2^−ΔΔCt^ method. (d) The expression of Rictor in normal (NL), fibrosis (LF), and cirrhosis (LC) rat livers (*n* = 3, separately) were measured by western blotting and quantified by densitometric analysis. *P < 0.05, **P < 0.01, ***P < 0.01. ns, no significant.

We established rat models (*n* = 22) to mimic the pathological development of liver fibrosis by CCl_4_. The histopathological changes in the livers were visualized by H&E staining, and collagen deposition was assessed by Masson staining (Figure 1a). The qPCR results revealed that miR-192 was gradually decreased with the severity of liver fibrosis (Figure 1b). Unlike miR-192, our study revealed a progressive increase in Rictor at both the mRNA (Figure 1c) and the protein (Figure 1d) levels, with a notably significant upregulation observed at the protein level.

### 3.2 miR-192 and Rictor were dysregulated in TGF-β1-activated HSCs

**Figure 2:**
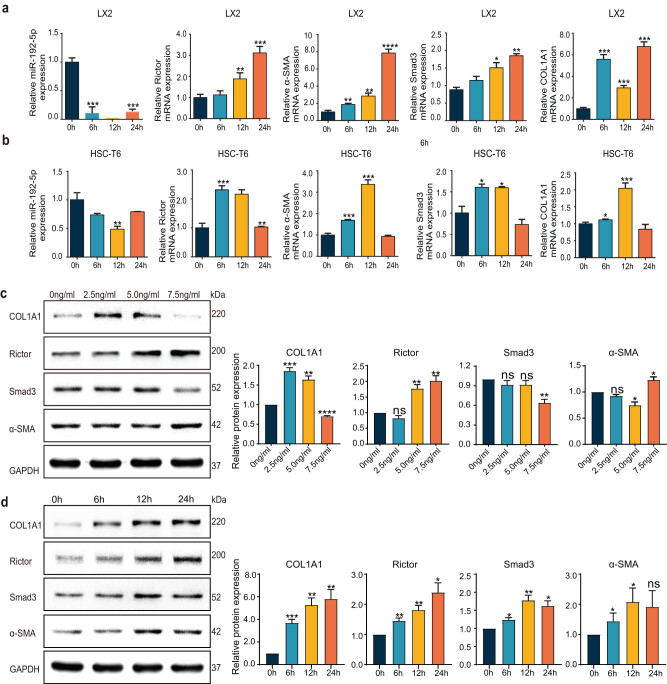
miR-192 and Rictor were dysregulated in TGFβ1-activated hepatic stellate cells: LX-2 (a) and HSC-T6 (b) cells were treated with 5 ng/mL TGF-β1 for 0, 6, 12, and 24 h. The expression of miR-192, α-SMA, Smad3, COL1A1, and Rictor was detected by qRT-PCR. The protein expression of COL1A1, Smad3, α-SMA, and Rictor was detected by western blotting in LX-2 cells and quantified by densitometric analysis. LX-2 cells were treated with 0, 2.5, 5.0, and 7.5 ng/mL TGF-β1 for 24 h (c) or with 5 ng/mL TGF-β1 for 0, 6, 12, and 24 h (d). Control was set to 1. *P < 0.05, **P < 0.01, ***P < 0.001. ns, no significant.

**Figure A1:**
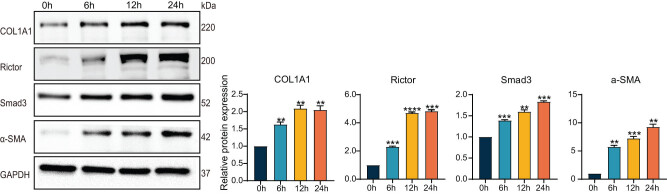
Fibrotic related genes and Rictor were dysregulated in TGFβ1-activated HSC-T6 cells. HSC-T6 cells were treated with 5 ng/mL TGF-β1 for 0, 6, 12, and 24 h.The protein expression of COL1A1, Smad3, α-SMA, and Rictor were detected by western blotting in HSC-T6 cells (left) and quantified by densitometric analysis (right).

The recombinant active human TGF-β1 protein was used to stimulate LX-2 cells to mimic the activation of HSCs. miR-192 was enriched in LX-2 cells, but its levels decreased significantly after the treatment of 5.0 ng/mL TGF-β. Conversely, the mRNA expression of α-SMA, Smad3, COLIA1, and Rictor exhibited a substantial increase (Figure 2a). The same trend was also observed in HSC-T6 (Figure 2b and Figure A1). Subsequently, we detected the relevant proteins in LX-2 cells activated by TGF-β1 (Figure 2c and d). Rictor and all fibrotic proteins exhibited time-dependent upregulation. In the context of varying TGF-β1 concentrations, although the expression of COLIA1 and Smad3 was not strictly upregulated by the TGF-β1, the expression of Rictor matched this concentration-dependent trend, peaking alongside α-SMA at TGF-β1 concentration of 7.5 ng/mL. Thus, we determined that miR-192 and Rictor were dysregulated during the process of HSC activation.

### 3.3 miR-192 exerted its anti-fibrosis effect in LX-2 by targeting Rictor

**Figure 3:**
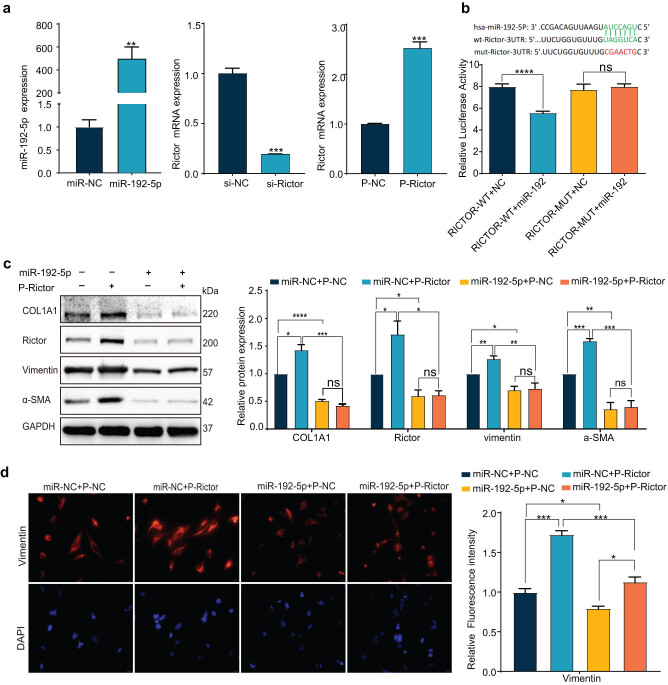
miR-192 exerted its anti-fibrosis effect in LX-2 by targeting Rictor. (a) The transfection efficiency of miR-192-5p mimics, si-Rictor, and plasmid-Rictor in LX-2 cells. (b) The binding site of miR-192 at the 3′UTR of Rictor was predicted by TargetScan. Luciferase reporter assay of LX-2 cells transfected with pmirGLO vectors carrying RICTOR wildtype or mutant-binding site together with miR-192-5p mimics or miR-NC mimic. (c) After co-transfection of miR-192-5p mimics/miR-NC and plasmid-Rictor/plasmid-NC, the relative proteins (COL1A1, Rictor, Vimentin, and α-SMA) were measured by western blotting and quantified by densitometric analysis. (d) The expression of Vimentin was detected by immunofluorescence and quantified by densitometric analysis. (Red: Vimentin; blue: DAPI; 500×. Control was set to 1.) *P < 0.05; **P < 0.01; ***P < 0.001; ****P < 0.0001. ns, no significant.

The transfection efficiency of miR-192-5p mimics, si-Rictor and plasmid-Rictor was verified in LX-2 cells (Figure 3a). The bioinformatic analysis by TargetScan (http://www.targetscan.org/) predicted a conserved binding site of miR-192 at the 3′UTR of Rictor with a high possibility ranking. A luciferase reporter assay showed that miR-192 distinctly inhibited the luciferase activity in the reporter vector containing wild-type 3′UTR of Rictor but not in others, confirming the direct targeting of miR-192 on Rictor (Figure 3b). Correspondingly, the expression of COL1A1 and α-SMA, as HSC activation markers, elevated after the overexpression of Rictor, whose function was almost counteracted by the co-transfection of miR-192 (Figure 3c). Moreover, Vimentin, a crucial cytoskeletal component, exhibited a positive correlation with Rictor expression but was notably impaired by the rescue of miR-192 (Figure 3d). Thus, we determined that miR-192 has the potential to preclude HSC activation by targeting Rictor.

### 3.4 High miR-192/low Rictor expression is associated with attenuation of the fibrotic-related genes expression by AKT/mTORC2 signaling pathway

**Figure 4:**
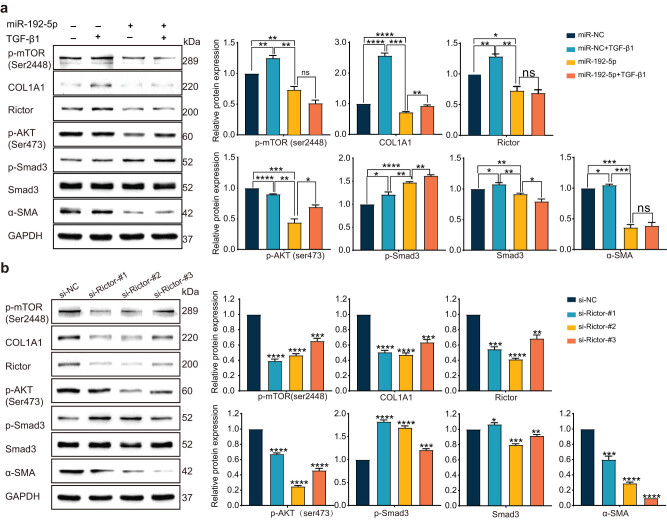
High miR-192/low Rictor expression is associated with attenuation of the fibrotic related genes expression by AKT/mTORC2 signaling pathway: LX-2 cells were transfected with miR-192-5p mimics/miR-NC for 24 h and cultured with/without 5 ng/mL TGF-β1 for another 24 h (a) or transfected with si-Rictor/si-NC for 48 h (b). The expression of phospho-mTOR (Ser2448), phospho-AKT (Ser473), COL1A1, phospho-Smad3, Smad3, α-SMA, and Rictor was detected by western blotting and quantified by densitometric analysis. Control was set to 1. *P < 0.05; **P < 0.01; ***P < 0.001; ****P < 0.0001. ns, no significant.

Since miR-192 and Rictor could regulate the fibrogenic phenotype of LX-2, the mechanism of how these molecules affect the development of LX-2 activation was subsequently investigated. miR-192-5p mimics and si-Rictor were separately transfected into LX-2 cells. Upregulation of miR-192 or downregulation of Rictor could significantly attenuate the expression of fibrogenic proteins and p-AKT (Ser473) and p-mTOR (Ser2448) in the AKT/mTORC2 signaling pathway (Figure 4a and b). However, p-Smad3, the representative molecule of the classic Smad signaling pathway was elevated in high miR-192 or low Rictor LX-2 cells. In the co-treated group with miR-192 and TGF-β1, we observed that the addition of miR-192 almost completely hindered the effects caused by TGF-β1.

To sum up, our results indicate that when Rictor was downregulated exogenously, whether by siRNA oligos or targeted inhibition of miR-192, the activated HSC exhibited a notable weakening of fibroblastic characteristics, which was reflected in the reduction of both the myofibroblastic protein markers and associated product synthesis. This demonstrated that miR-192 could at least partially inhibit the TGF-related activation pathway in HSCs, thereby inhibiting the occurrence and development of liver fibrosis by targeting Rictor.

## 4 Discussion

**Figure 5:**
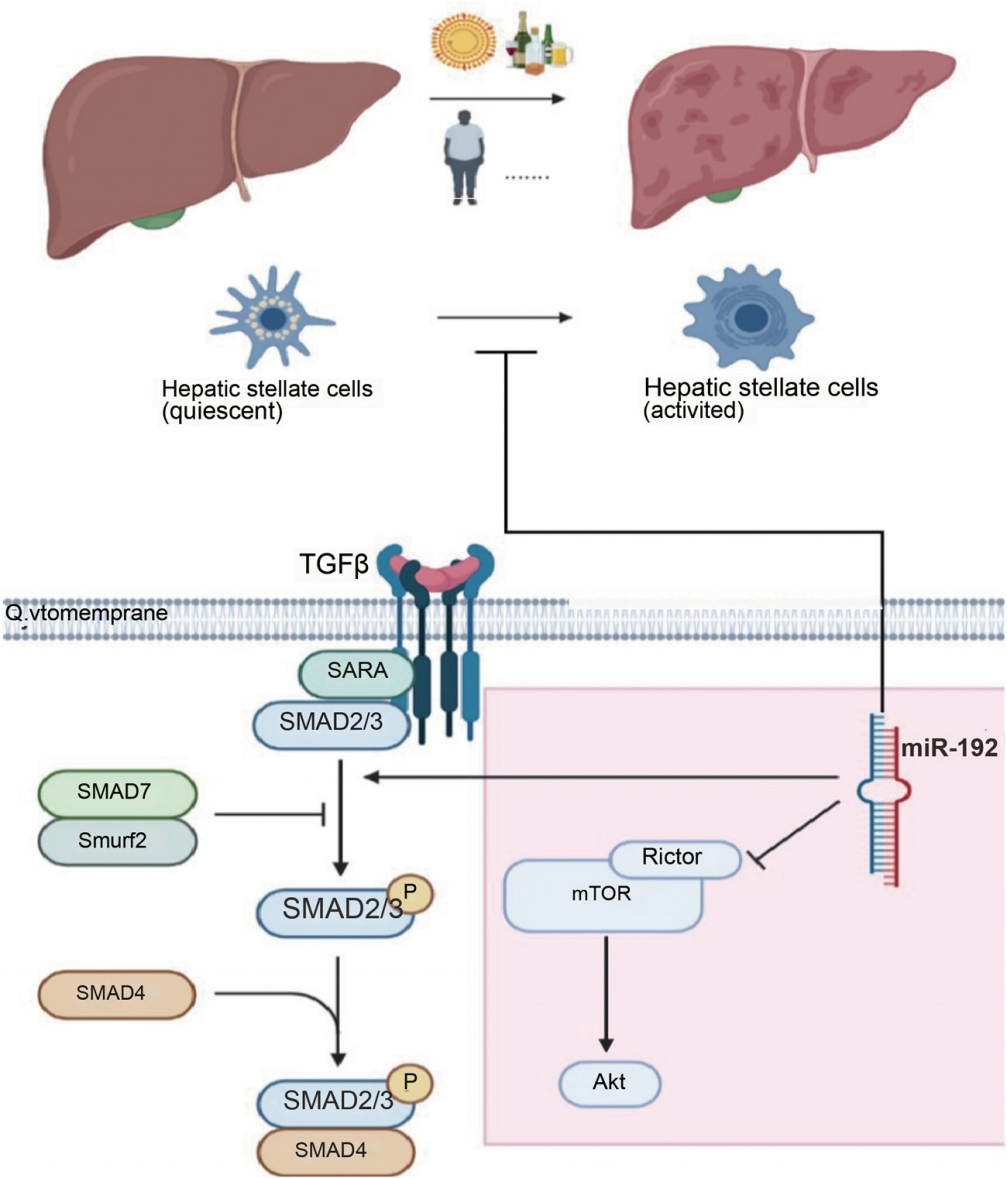
miR-192 could inhibit the activation of hepatic stellate cells by targeting Rictor, impeding TGF-β pathway by AKT/mTOR signaling (in the red areas). The Figure was created in BioRender.com.

In this research, we proved that miR-192 could effectively inhibit the activation of HSCs by targeting Rictor, which disrupts the TGF-β pathway through the AKT/mTOR signaling cascade (Figure 5). We observed a consistent pattern where miR-192 was downregulated, and Rictor was upregulated in CCl_4_-induced rat fibrotic liver models. Furthermore, our research demonstrated the negative regulatory role of miR-192 in HSC activation through its interaction with Rictor in LX-2 cells. These findings offer a novel target for the fibrosis treatment.

Liver fibrosis is a pathological protective response to various causes leading to liver injury [5]. It could potentially progress into cirrhosis, and without effective treatment, it may lead to liver failure and liver cancer. However, liver fibrosis is a dynamic, bidirectional process that has an inherent capacity for recovery and remodeling [22]. Therefore, it is meaningful to research on these targets, which could inhibit the occurrence and development of fibrosis.

The function of miR-192 was first reported in renal pathology, and whether it was pro-fibrotic or anti-fibrotic had been controversial over the past decade [23]. Unlike the pleiotropic roles in the kidney, it was universally accepted that miR-192 was a signal of liver health condition. The upregulation of serum miR-192 is identified as a biomarker of several liver diseases [24,25,26,27]. Conversely, its downregulation was reported in both hepatocytes [28] after acute liver injury and HSCs, the major source of miR-192 in healthy liver [17]. Although one recent research reported that exosomal transferred miR-192 was secreted and transmitted from JFH-1 (HCV genotype 2a) stably replicating Huh-7 cells into HSCs, and upregulated fibrogenic markers in HSCs [29], our results demonstrated that miR-192 was downregulated in the activation of HSCs, and the regain of miR-192 notably restrained the fibrotic markers of HSCs in both RNA and protein levels. These discordances might be owing to differences in cell types, etiologic factors, and methods of HSC activation and deserve further investigation.

Rictor was a key component of mTORC2. It was reported that the most prominent function of mTORC2/Rictor was phosphorylating Akt/protein kinase B at Ser473, thereby promoting cell survival, proliferation, and growth [10]. mTORC2/Rictor was also involved in the regulation of ion transport, cell survival, proliferation, cytoskeleton remodeling, and migration by phosphorylating other members of the AGC (PKA/PKG/PKC/SGK1) protein kinase family [9,30]. Zhou et al. testified that miR-185 could inhibit fibrogenic activation of HSC by Rictor recently [15]. However, we demonstrated that miR-192 could also exert anti-fibrosis function by targeting the mTORC2/Rictor pathway to inhibit the activation of HSCs. These two conclusions are not contradictory, since it is rational that the same molecule might be regulated by different miRNAs. Instead, this phenomenon suggests that Rictor has the potential to be a node and quite an important molecule in HSC activation.

Among the existing three isoforms of TGF-β, β1 was the most quantitatively important and widely investigated one in liver fibrogenesis [31]. It is now well accepted that after binding to its receptors, TGF-β1 can exert its biological effects through the activation of its downstream mediators, Smad2 and Smad3, by phosphorylation [32]. In renal fibrosis, interestingly, although Smad2 shares a high degree of structural similarity to Smad3, overexpression of Smad2 attenuated TGF-β1-induced Smad3 phosphorylation and collagen I matrix, indicating that Smad2 works antagonistically to Smad3 in the progression of renal fibrosis [33]. Coincidentally, it is also reported in liver fibrosis [34]. Thus, we selected Smad3 and p-smad3 as indicators of HSC activation through the TGF pathway. However, a paradox emerged when we observed that after transfection with the miR-192-5p mimic and si-Rictor, Smad3 expression decreased, while its activated form, p-Smad3, exhibited the opposite trend. Although p-Smad3, an indicator of the TGF-β pathway, was increased, the effects of the TGF-β pathway were actually inhibited by miR-192. This seemingly contradictory outcome can be rationalized by the multifaceted nature of TGF-β signaling. In addition to the classical Smad pathway, mTOR, a target of the Akt kinase, plays an important role in the PI3K–Akt pathway and contributes to TGF-β-induced EMT through non-Smad signaling pathway [8]. These observations are consistent with our findings and underscore the importance of non-Smad pathways, as they can even impede the dominant Smad pathway in some circumstances. This intricate network of signaling pathways raises compelling questions: how do miR-192 and Rictor affect the Smad pathway? How do non-Smad and Smad pathways interact with each other? And what is the potential of miR-192 in impeding liver fibrosis *in vivo*? These critical questions underscore the need for further research.

In conclusion, we determined that miR-192 could inhibit the activation of HSCs by directly targeting Rictor within the AKT/mTORC2 signaling pathway. Herein, we provided a new standpoint to the activation of HSCs and some potential targets for the therapy of liver fibrosis.
